# Gender-Based Clinical, Therapeutic Strategies and Prognosis Differences in Atrial Fibrillation

**DOI:** 10.3390/jcdd10100434

**Published:** 2023-10-18

**Authors:** Aurelio Quesada, Javier Quesada-Ocete, Blanca Quesada-Ocete, Víctor del Moral-Ronda, Javier Jiménez-Bello, Ricardo Rubini-Costa, Carl J. Lavie, Daniel P. Morin, Fernando de la Guía-Galipienso, Ricardo Rubini-Puig, Fabian Sanchis-Gomar

**Affiliations:** 1 Arrhythmia Unit, Cardiology Service, General University Hospital Consortium of Valencia, 46014 Valencia, Spain; javierquesadaocete@gmail.com (J.Q.-O.); blanca.quesada.ocete@gmail.com (B.Q.-O.); javijibel@gmail.com (J.J.-B.); 2 School of Medicine, Catholic University of Valencia San Vicente Mártir, 46001 Valencia, Spain; ricardo.rubini93@gmail.com (R.R.-C.); fdelaguia@gmail.com (F.d.l.G.-G.); rrpuig@gmail.com (R.R.-P.); 3 Department of Cardiology, Hospital Universitario de Tarragona Joan XXVIII, 43005 Tarragona, Spain; moral.ronda@gmail.com; 4 Hospital IMED, 46100 Valencia, Spain; 5 John Ochsner Heart and Vascular Institute, Ochsner Clinical School, The University of Queensland School of Medicine, New Orleans, LA 70121, USA; clavie@ochsner.org (C.J.L.); dmorin@ochsner.org (D.P.M.); 6 Glorieta Policlinic, 03700 Denia, Spain; 7 Cardiology Service, Hospital HCB Benidorm, 03501 Benidorm, Spain; 8 Emergency Room Department, General University Hospital Consortium of Valencia, 46014 Valencia, Spain; 9 Division of Cardiovascular Medicine, Stanford University School of Medicine, Stanford, CA 94305, USA

**Keywords:** atrial fibrillation, gender, electrical cardioversion, heart failure, mortality

## Abstract

Background: There are limited data on gender-based differences in atrial fibrillation (AF) treatment and prognosis. We aimed to examine gender-related differences in medical attention in an emergency department (ED) and follow-up (FU) among patients diagnosed with an AF episode and to determine whether there are gender-related differences in clinical characteristics, therapeutic strategies, and long-term adverse events in this population. Methods: We performed a retrospective observational study of patients who presented to a tertiary hospital ER for AF from 2010 to 2015, with a minimum FU of one year. Data on medical attention received, mortality, and other adverse outcomes were collected and analyzed. Results: Among the 2013 patients selected, 1232 (60%) were female. Women were less likely than men to be evaluated by a cardiologist during the ED visit (11.5% vs. 16.6%, *p* = 0.001) and were less likely to be admitted (5.9% vs. 9.5%, *p* < 0.05). Electrical cardioversion was performed more frequently in men, both during the first episode (3.4% vs. 1.2%, *p* = 0.001) and during FU (15.9% vs. 10.6%, *p* < 0.001), despite a lower AF recurrence rate in women (9.9% vs. 18.1%). During FU, women had more hospitalizations for heart failure (26.2% vs. 16.1%, *p* < 0.001). Conclusions: In patients with AF, although there were no gender differences in mortality, there were significant differences in clinical outcomes, medical attention received, and therapeutic strategies. Women underwent fewer attempts at cardioversion, had a lower probability of being evaluated by cardiologists, and showed a higher probability of hospitalization for heart failure. Being alert to these inequities should facilitate the adoption of measures to correct them.

## 1. Introduction

Atrial fibrillation (AF) is the most common cardiac arrhythmia, with a worldwide prevalence of ~38 million in 2017, and is associated with high mortality and morbidity rates [1]. In adjusted models, AF is associated with increased morbidity (especially stroke and heart failure) and mortality [1]. From a pathophysiological, epidemiological, diagnostic, and management perspective, the influence of gender on cardiovascular disease has been widely studied and described [2]. In this regard, AF is not an exception, showing gender variations.

AF is more frequent in men than in women, with a global annual incidence per year of 77.5 cases per 100,000 population and 48.7 cases per 100,000 population, respectively [3]. It is well known that AF has a more significant impact on women’s morbidity and mortality. Compared to those without AF, women with AF have an approximately three-fold higher risk of suffering a cardiovascular disease (CVD)-related event (i.e., hospitalization or death) related to arrhythmia. Men have a 1.8-fold higher risk than the general male population [4].

Female sex is an independent risk factor for stroke [5]. In the setting of AF, women have up to 1.3-fold higher risk of stroke and systemic embolism compared with men, even if they are treated with non-vitamin K antagonist oral anticoagulants (NOACs), and especially in those older than 75 years [6,7,8]. Some authors have suggested gender-based differences in AF therapy. For example, it has been previously reported that conservative treatment is more frequently recommended for women among patients showing atypical or asymptomatic presentations. At the same time, rhythm control is more likely to be pursued in men [7,8]. Kloosterman et al. reported that men are more likely to be treated with electrical cardioversion (ECV) and/or NOAC therapy. At the same time, women are more likely to be treated with only ß-blockers or calcium antagonists [9]. Catheter ablation for AF has also been studied, showing that it is a more frequently used strategy in men despite a similar benefit on quality-of-life scales between men and women [9]. In women, systemic anticoagulation is used less often, and when administered, the therapy may be of lower quality, although the benefits of anticoagulation are higher in women [4,6,8].

Therefore, we compared the medical attention characteristics (primary objective), clinical course, morbidity, and mortality of patients who attended an ED for an AF episode (index episode), stratified by gender.

## 2. Materials and Methods

### 2.1. Study Design and Population

**Figure 1:**
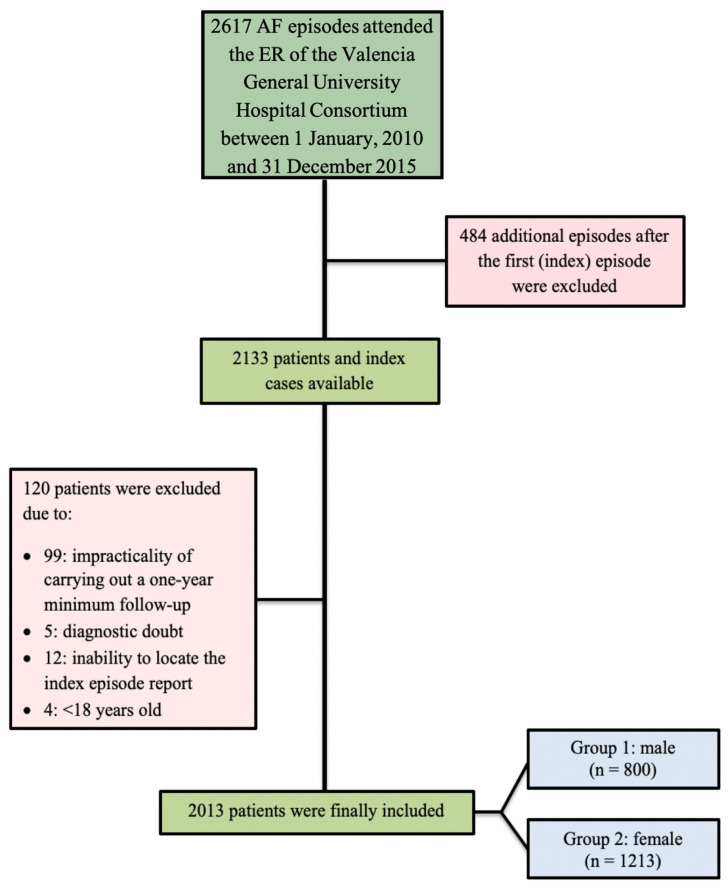
Flow chart representing the total sample after applying the inclusion and exclusion criteria. AF = atrial fibrillation; ER = emergency room.

We conducted a retrospective observational study of consecutive patients obtained from a database consisting of 2617 AF episodes who presented to the emergency department (ED) of the Valencia General University Hospital Consortium (Consorcio Hospital General Universitario de Valencia—CHGUV) with a final diagnosis of AF between 1 January 2010 and 31 December 2015. We captured all AF presentations during the study period. After eliminating repeated episodes and applying the exclusion criteria, 2013 patients were included. Two study groups were finally obtained by dividing the patients according to gender: women and men. Data from patients over 18 years old who presented with an electrocardiographic (ECG) diagnosis of AF during their admission to the ED were recorded (Figure 1). Inclusion in the study required at least one year of follow-up. Patients were excluded in case of a mistake in the AF diagnostic, inability to locate the index episode report, further AF episode/s suffered after the AF index episode, and follow-up loss. The Clinical Research Ethics Committee of the CHGUV approved the study.

### 2.2. Variables Collected

Age and gender were collected for demographics. Physiological variables included heart rate (HR), blood pressure (BP), oxygen saturation, and left ventricular ejection fraction (LVEF). Likewise, clinical data were collected, including CVD risk factors, previous structural heart disease, other cardiological history, and other comorbidities. Characteristics of the index episode, blood test results, clinical presentation (asymptomatic, palpitations, HF, etc.), treatment administered in ED (drugs, cardioversion, etc.), spontaneous conversion to sinus rhythm, assessment by cardiology services, and patient destination after the episode were also collected.

### 2.3. Follow-Up

A minimum of one year follow-up was performed after the index episode by accessing the electronic medical record and/or telephonic call. The following data were collected: all-cause mortality, first arrhythmia-related presentation to ED, first HF-related presentation to ED or hospitalization, first recurrence of AF, minor hemorrhagic events, major hemorrhagic events (defined as gastrointestinal, intracranial, pulmonary, or retroperitoneal bleeding), embolic events, type of embolism (i.e., ischemic stroke, transient ischemic attack (TIA), or peripheral embolism), first CV after the index episode (date and effectiveness), as well as pharmacological treatment administered.

### 2.4. Statistical Analysis

Quantitative variables are expressed as mean ± standard deviation (SD), and qualitative values are presented as percentages. The Chi-square test was used to compare qualitative variables, and Student’s *t*-test was used for quantitative variables. The cumulative event-free survival probability was estimated using the Kaplan–Meier (KM) method and compared using the log-rank test and calculating the hazard ratio (HR). Multivariable Cox linear regression analyses were performed for HF-related hospitalizations and all-cause mortality to test the independent relationship between gender and other clinical variables. Variables were selected based on previous research evidence and the authors’ clinical experience. The results of all statistical analyses were considered statistically significant when *p* < 0.05. All data were analyzed using the IBM SPSS statistical package v25.0 or GraphPad Prism v8.0.

## 3. Results

### 3.1. Baseline Characteristics of the Study Population

A total of 1213 (60.3%) female and 800 (39.7%) male patients were followed up for a mean period of 3.3 ± 1.5 years. The average age of participants was 75 ± 13 years (60.3% above 75 years; 23% above 85 years). Among the risk factors for CVD, the most frequently observed were hypertension (HTN), dyslipidemia, and type 2 diabetes mellitus (T2DM), showing a prevalence of 76%, 39.8%, and 36.5%, respectively. The prevalence of smoking and chronic kidney disease (CKD) was low, at approximately 10% for each (Table 1).

Table 1 also shows gender-based differences in the recorded variables. Women were older than men (77.8 ± 10.6 vs. 70.9 ± 14.1 years). Regarding clinical characteristics, the prevalence of CVD risk factors and other comorbidities was uneven between women and men. Men showed a higher prevalence of respiratory diseases, such as chronic obstructive pulmonary disease (COPD) or obstructive sleep apnea (OSA), as well as smoking and alcohol abuse. Specifically, we found significantly higher prevalence rates in men than in women for OSA (5.5% vs. 2.8%; *p* = 0.002), peripheral artery disease (PAD) (7.3% vs. 3.1%; *p* < 0.001), alcohol abuse (3.6% vs. 0.2%; *p* < 0.001), smoking (20.3% vs. 3.4%; *p* < 0.001), history of acute myocardial infarction (10.6% vs. 6%; *p* < 0.001) and/or ischemic cardiomyopathy, and dilated cardiomyopathy (DCM; 6.1% vs. 1.8%; *p* < 0.001). In contrast, the prevalence of HTN and obesity were significantly higher in women (80.7% vs. 68.9%; *p* < 0.001 and 9.6% vs. 6.9%; *p* = 0.034, respectively). Of note, the prevalence of HF was significantly higher among women (22.3% vs. 16.3%), as was the prevalence of mitral valve disease (12.6% vs. 8.5%). Baseline differences were also translated into prognostic score differences, with a higher average CHA_2_DS_2_-VASc score in women than in men (4.2 ± 1.4 vs. 2.7 ± 1.6, *p* < 0.001). No significant difference between the genders was found in anticoagulation therapy or previous ECV.

### 3.2. Gender-Based Clinical Differences at Index Episode

As Table 2 shows, asymptomatic presentation was more frequent in men, while various symptoms and signs were significantly more frequent in women: palpitations (25.3% vs. 33.5%; *p* < 0.001), HF (26.1% vs. 33.1%; *p* = 0.001), and acute pulmonary edema (3.1% vs. 5%; *p* = 0.039). Although a statistically significant difference was found in HR at admission, 122 ± 31 beats per minute (bpm) in women and 118 ± 35 bpm in men (*p* = 0.002), it was clinically irrelevant.

### 3.3. Healthcare Resource Use and Therapeutic Differences

The use of healthcare resources and therapeutic differences were analyzed during the index episode and subsequent follow-up. A significant gender-based difference was found in the proportion of patients being evaluated by the cardiologist during the first episode: 16.6% of males and 11.5% of females were visited by a cardiologist (*p* = 0.05).

**Figure 2:**
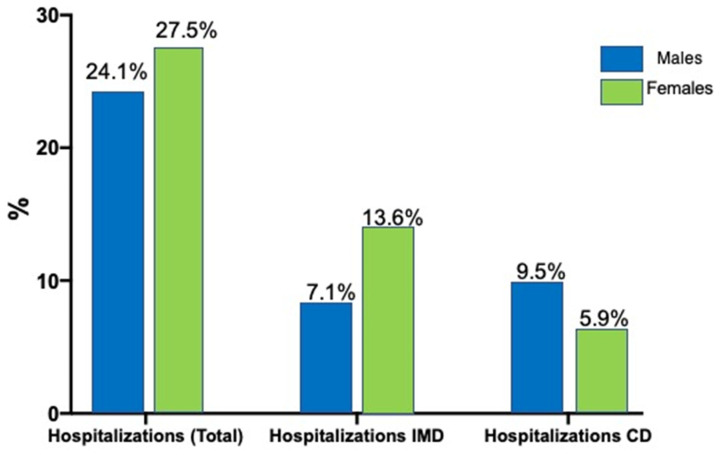
Total hospitalization percentage of index episodes. IMD = internal medicine department; CD = cardiology department.

Significant differences were also found in the number of patients transferred to other medical services: 7.1% of men and 13.6% of women (approximately half of those who were admitted to ER) were transferred to internal medicine service (*p* < 0.001). In comparison, only 5.9% of women were transferred to the cardiology service versus 9.5% of men (*p* < 0.05) (Figure 2).

Furthermore, differences were found in the prescribed treatment for preventing cardioembolic events following hospital or ER discharge. Among women, 45.5% were prescribed OACs, an absolute difference of 5.1% more often than men (*p* < 0.05). Regarding anti-arrhythmic treatment at discharge from the ED, women were more frequently prescribed digoxin (8.62% vs 13.02%, *p* < 0.01). There were no significant differences in the remaining antiarrhythmic drugs (Tables S1 and S2 of Supplementary Data).

**Figure 3:**
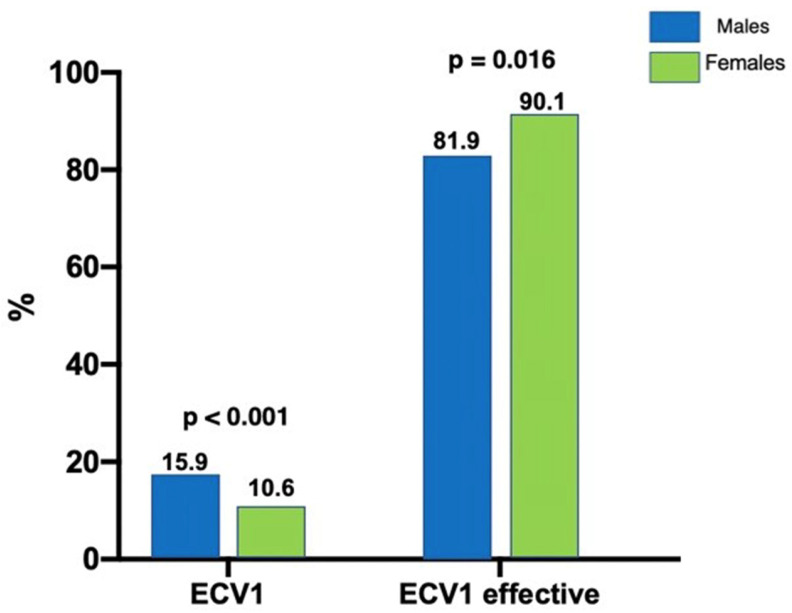
Percentage of patients with AF who underwent electrical cardioversion (ECV) and effectiveness by gender. ECV1 = first ECV.

Moreover, we found significant gender differences in the frequency at which ECV was used. During the index episode, men were more frequently treated with ECV (3.4% vs. 1.2%; *p* = 0.001). During the follow-up period, as Figure 3 shows, ECV was performed in only 10.6% of women versus 15.9% of men (*p* < 0.001), with a higher recurrence rate after the first ECV in men (23 of 127, 18.12%) than in women (12 of 128, 9.94%; *p* = 0.016).

### 3.4. Comparison of Long-Term Adverse Events

**Figure 4:**
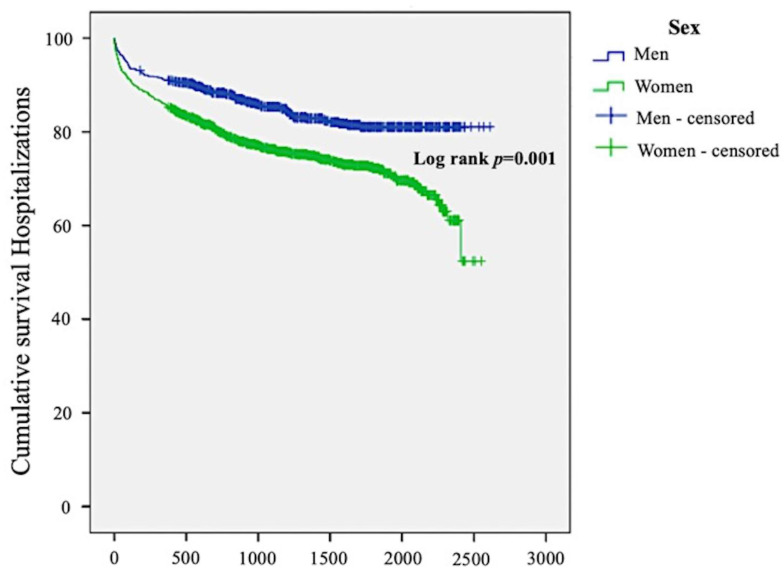
Kaplan–Meier survival analysis curves for HF-related hospitalization by gender. HF = heart failure.

During the follow-up period, significant gender differences were observed in the frequency of HF occurrence. Women were more prone than men to require hospitalization for HF (26.2% vs. 16.1%; *p* < 0.001). Likewise, women with AF were admitted to the hospital due to HF with a higher frequency than men (33.1% vs. 26.1%; *p* = 0.001; Figure 4).

**Figure 5:**
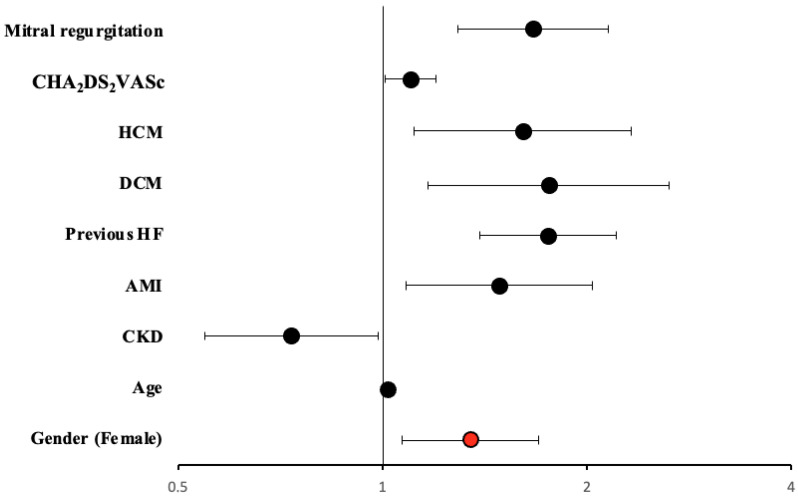
Hazard ratio and 95% CI in multivariate Cox regression analysis for HF-related hospitalizations. Marked in red, female gender has an independent effect on this end-point. Abbreviations: AMI = acute myocardial infarction; CKD = chronic kidney disease; CI = confidence interval; DCM = dilated cardiomyopathy; HCM = hypertrophic cardiomyopathy; HF = heart failure; HR = hazard ratio; HTN = arterial hypertension; T2DM = type 2 diabetes mellitus.

We performed a multivariate analysis to determine whether female gender was an independent risk factor for HF-related hospitalizations among patients with AF. Variables included were age, gender, HTN, T2DM, CKD, COPD, peripheral arteriopathy, acute myocardial infarction (AMI), previous HF, and dilated cardiomyopathy (DCM). Table 3 shows that female patients with AF were independently associated with the occurrence of hospitalizations due to HF. The other independent risk factors for this event were age and other comorbidities such as T2DM, CKD, COPD, previous AMI, HF, and DCM (Figure 5).

There was no gender difference in the frequency of recurrence of AF (46% vs. 45.7%; *p* = 0.962), nor in mortality (39% vs. 41.6%; *p* = 0.239), incidence of hospitalization (30.3% vs. 32%; *p* = 0.411), emergency care (55.5% vs. 59.1%; *p* = 0.109), ED admissions (27.5% vs. 24.1%; *p* = 0.109), embolism (7.3% vs. 8.6%; *p* = 0.285), or major or minor bleeding (7.8% vs. 5.9%; *p* = 0.094, and 8% vs. 8.1%; *p* = 0.949, respectively).

### 3.5. Multivariate Analysis of Mortality

**Figure 6:**
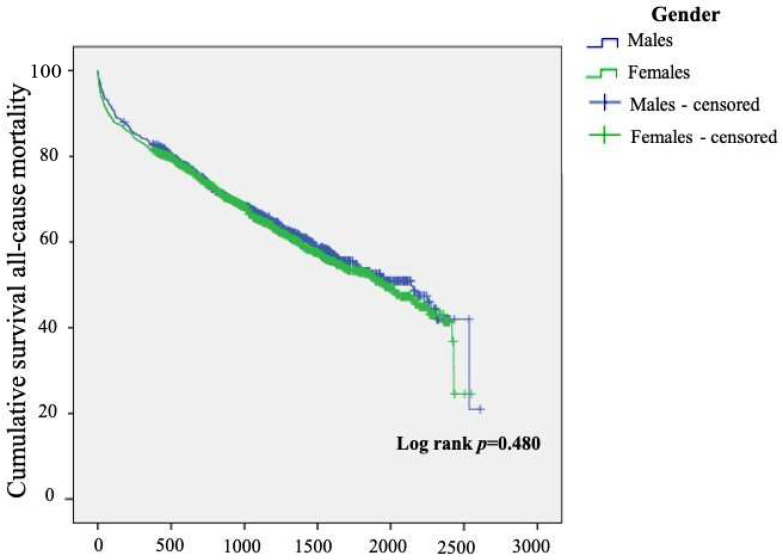
Kaplan–Meier survival analysis curve for all-cause mortality.

Over an average of 3.3 ± 1.5 years of follow-up, there were 817 deaths, including 312 out of 800 men (39%) and 505 out of 1213 women (41.6%). Figure 6 displays survival over time, stratified by gender.

**Figure 7:**
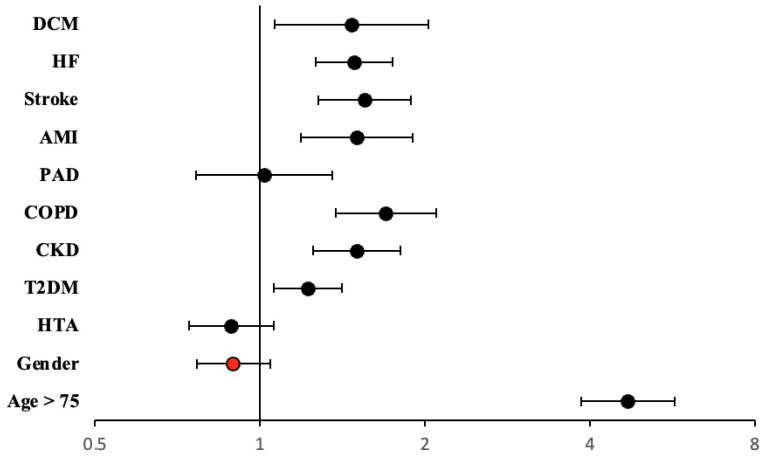
Hazard ratio and 95% CI in multivariate Cox regression analysis on all-cause mortality. Marked with a red circle, female gender has an independent effect on this end-point. Abbreviations: AMI = acute myocardial infarction; CKD = chronic kidney disease; CI = confidence interval; COPD = chronic obstructive pulmonary disease; DCM = dilated cardiomyopathy; HF = heart failure; HR = hazard ratio; HTN = arterial hypertension; PAD = peripheral artery disease; T2DM = type 2 diabetes mellitus.

We also performed a multivariate analysis to determine whether female gender was an independent risk factor for mortality among patients with AF. Variables included were age, gender, HTN, T2DM, CKD, COPD, peripheral arteriopathy, acute myocardial infarction (AMI), stroke, HF, and dilated cardiomyopathy (DCM). Table 4 shows that female patients with AF were not independently associated with all-cause mortality. However, this event was independently associated with age and other comorbidities such as T2DM, CKD, COPD, previous AMI, HF, and DCM (Figure 7).

## 4. Discussion

Our study shows relevant clinical use of health resources and prognosis gender-related differences in patients with AF. In a 5-year series of consecutive patients who attended the emergency department for PA, the male-to-female ratio was 2:3. Women experienced more symptomatic AF episodes and received different therapies than their male counterparts. Likewise, women were less likely to be evaluated by ER cardiologists and less frequently transferred to the cardiology service during their index episodes.

The higher number of women than men in our study is somewhat surprising. Most epidemiological studies report a higher prevalence of AF in men [10,11,12,13]. Apparent differences in design from ours (population vs. cohort studies) may explain this finding. Still, given that a higher proportion of females has also been found in other extensive studies of AF patients [14], it would be worthwhile not to rule out other sources of bias influencing these discrepancies.

However, one of our most relevant findings has been the detection of important differences in the assistance received by women compared to men. These differences have also been noted in the few studies that have evaluated this issue. Thus, in a previous study, Bhave et al. [14] reported gender-related differences in healthcare services provided to AF patients and concluded that men receive more aggressive care. Specifically, women were less likely to be visited by a cardiologist or electrophysiologist, undergo catheter ablation procedures, or be prescribed oral anticoagulants, rate control, and antiarrhythmic drugs. The difference between the study conducted by Bhave et al. and ours was that we analyzed data registered during acute care in the emergency care setting. In contrast, they studied the management received after the initial AF diagnosis. Hence, our findings reinforce and expand on the evidence provided by this study. In addition, similarly to our results, Potpara et al. [15] reported that women with AF were more symptomatic than men. This fact was also confirmed by Rienstra et al. [16], who concluded that patients with asymptomatic AF were more often men. Another recent study [17] found, as in our study, that rhythm control strategies were less frequent than in men, even though women had poorer perceived quality of life and were more symptomatic.

In our study, ECV was less frequently performed for women. This finding concurs with Andrade et al. [4] and Kloosterman et al. [9], who reported that ECV was more frequently used in men in previous studies. Likewise, Alegret et al. [18] also reported that gender was an important determinant in deciding the treatment for managing patients with AF (i.e., rhythm control strategy or rate control only) in 67 Spanish hospitals during the last decade. These authors observed a lower percentage of women undergoing ECV, and this difference was particularly significant among older asymptomatic patients. Kloosterman and colleagues [9] described demographic and clinical gender-related differences similar to ours. Despite a slightly longer hospital stay and a higher rate of minor bleeding among women, the post-ECV improvement in quality of life and cognitive status found on various scales was similar between the two genders [4,9].

To highlight, unlike what has been reported [19,20], we found that scheduled cardioversions during follow-up had lower recurrence rates in women than in men. This should support efforts for a more generalized use in females, as opposed to what we have observed (see below).

We also found a higher hospital admission rate for HF in women with AF during the follow-up period. Although they did not evaluate long-term HF admissions, Piccini et al. [8] described a similar frequency difference between the genders in the occurrence of new HF during follow-up. Although it was not statistically significant, O’Neal et al. [21] also reported a numerically increased risk of hospitalization in women with AF. Undoubtedly, the higher mean age of the women in our population may have influenced our findings and the higher baseline rates of HF among women. However, this might be explained by the gender-related differences in therapy received [22]. In this regard, Bhave et al. [14] also reported less aggressive management and treatment of AF in women, which might ultimately influence disease evolution with poorer results. In addition, Cheng and Kong [7] found gender-based differences in the efficacy of medical management of patients with AF. Compared with men, women who were not anticoagulated had greater thromboembolic risk but benefited from more significant risk reduction when systemically anticoagulated.

We found no significant gender-related difference in mortality among these patients with AF. Marijon et al. [23] investigated which factors were associated with mortality and several specific causes of death, finding neutral results regarding the influence of gender. Moreover, Potpara et al. [15] found no significant difference between male and female patients regarding all-cause mortality, CVD death, or sudden cardiac death. Other studies, however, associated female gender with higher mortality rates in patients with AF [7,14,22,24,25,26]. The clinical guideline of the European Society of Cardiology states that, while men with AF have a mortality 1.5 times higher than men without AF, similarly afflicted women have a mortality two times that of their counterparts without AF [24]. However, these differences decreased markedly when the study was carried out in developed countries, a distinction which must also be considered in our study [3]. In contrast, Piccini et al. [8] found higher mortality from all causes in men AF patients.

The higher mortality in women described by other studies may be due to the higher embolic risk in women since mortality differences decrease in effectively anticoagulated populations. This difference in all-cause mortality reduces significantly, as we also observed in our study, and the leading cause of death becomes CVD death [23].

### 4.1. Possible Mechanisms Involved

Indeed, many biological differences could explain the differences that we found. Some studies suggest that estrogens reduce AF prevalence in premenopausal women by decreasing overall CVD risk associated with a better lipid profile and inducing prolongation of the atrial action potential [27]. Higher testosterone levels elicit the opposite effects in men, leading to more fibrotic and proarrhythmic myocardial remodeling [28]. On the other hand, when comparing older patients of both sexes, this hormonal benefit disappears progressively, and other comorbidities develop a higher prevalence. Additionally, electroanatomical mapping during pulmonary vein ablation procedures has found a higher percentage of low-voltage areas in the left atrium in women, suggesting increased fibrosis and cardiomyocyte dysfunction [29,30]. This could predispose older women to AF and other clinical repercussions, such as left ventricle hypertrophy, diastolic HF, and valve disease [28], and would partly explain the existence in our series (with older age) of a higher percentage of women and the greater tendency to hospitalizations of cardiovascular cause found. However, it does not explain the lower prescription of cardioversion in women or the greater success of cardioversion, with a higher recurrence rate in men.

Given that the referred studies on atrial fibrosis have been performed mainly in Japan, it has been suggested that there could be ethnic and/or cultural differences among Caucasian patients [30]. Furthermore, sex-related differences in response to antiarrhythmic drugs could account for a higher rate of recurrences in men. These aspects require further studies to expand our understanding of the problem.

Finally, not only biological factors seem to be acting on our results. As mentioned above, cultural factors significantly influence AF patient care behaviors, both in our series and in other studies. It is important to highlight that women had a higher mean age and comorbidities; some works (in contrast to ours) report a slightly higher recurrence rate after ECV in women [19,20], a lower prevalence of symptomatic women with AF in some other studies has been reported [18] and, finally, female gender seems to increase the risk of complications (e.g., thromboembolism) following ECV of acute AF (duration <48 h), especially in patients aged >75 years and time to ECV exceeding 12 h [31]. All these aspects could influence the undertreatment of women with moderate-to-high risk and the underuse of ECV among women [18,32], as we observed in the present study. However, most of the evidence supports the opposite, either in general or in females in particular, and does not justify females receiving different assistance than males. Although the prognostic impact of the choice of strategy (rhythm vs. rate control) may still be subject to some controversy, the majority of evidence indicates that, with current treatments, the rhythm control strategy provides better symptomatic control and quality of life [33] as well as lower mortality [34]. Thus, the lower use of ECV in women (the first step in the rhythm control strategy), especially when we have found that it is associated with less recurrence of AF, has little scientific justification, and again raises suspicion of a gender bias.

We must be aware that the results of higher mortality observed in some studies, especially those carried out in less economically developed areas, or the greater rate of HF, a surrogate of mortality found in ours, may not reflect biological sex-related differences in mortality but rather gender-related differences in health care delivery. Being alert to these inequities should facilitate taking measures to correct them.

### 4.2. Study Limitations

Our data may be subjected to information biases due to varying levels of detail in the clinical reports, or some information needs to be included in the clinical history. Of note, if it was appropriate according to the antecedent diagnosis dates, we completed the collection of baseline information during the follow-up period. Regarding mortality, there were baseline differences in the characteristics and comorbidities of both groups that could affect the prognosis. A multivariate analysis was carried out to eliminate the effects of confounders, confirming that gender did not independently impact mortality rates in our population. Still, HF, more frequent in women, did. The fact that all patients were enrolled in the ER could be considered a source of selection bias. However, the sample was representative of the population.

Although the data relate to patients with AF between 2010 and 2015, our perception is that current practice and treatment strategies for AF have remained relatively similar compared to the years of study. However, this remains to be confirmed in future studies without the distortions imposed by the COVID-19 pandemic. Finally, other treatments for rhythm control, such as AF catheter ablation, were not evaluated.

## 5. Conclusions

There are significant gender-related differences in patients with AF. Although we did not find a significant independent gender-related difference in mortality in these patients, female gender increased the risk of admission for HF, a surrogate of mortality, in the long-term follow-up of patients with AF. Furthermore, women differed in unfavorable baseline characteristics compared to men, mainly in age and greater CHA_2_DS_2_-VASc score, and more frequently present with symptomatic AF. Despite these adverse profiles, there were marked healthcare differences according to gender, such as women being less likely to receive a cardiology consultation or to be transferred to the cardiology department. ECV was also used less frequently in women in the ER and during follow-up, notwithstanding its better results.

Finally, all of our findings related to the use of health resources and treatment strategies would point to the fact that differences in HF may be more related to a gender bias than a sex one. Recognizing these gender differences may help clinicians improve AF’s acute and chronic management.

## Figures and Tables

**Table 1:** Baseline characteristics of the study population by gender.

	Total (*n* = 2013)	Male (*n* = 800)	Female (*n* = 1213)	*p*-Value
Demographic data				
Age (years)	75 ± 12.6	70.86 ± 14.1	77.8 ± 10.6	<0.001
<65	369 (18.3%)	233 (29.1%)	136 (11.2%)	
65–74	430 (21.4%)	172 (21.5%)	258 (21.3%)	
75–84	744 (37%)	278 (34.8%)	466 (38.4%)	
>85	470 (23.3%)	117 (14.6%)	353 (29.1%)	
Comorbidities				
Hypertension	1530 (76%)	551 (68.9%)	979 (80.7%)	<0.001
T2DM	734 (36.46%)	273 (34.1%)	461 (38%)	0.077
Smoking	203 (10.1%)	162 (20.3%)	41 (3.4%)	<0.001
Obesity	171 (8.5%)	55 (6.9%)	116 (9.6%)	0.034
OSA	78 (3.9%)	44 (5.5%)	34 (2.8%)	0.002
Dyslipidemia	177 (8%)	288 (36%)	513 (42.3%)	0.005
Alcohol abuse	801 (39.8%)	29 (3.6%)	3 (0.2%)	<0.001
COPD	32 (1.6%)	130 (16.3%)	47 (3.9%)	<0.001
CKD	231 (11.5%)	92 (11.5%)	139 (11.5%)	0.978
PAD	96 (4.8%)	58 (7.3%)	38 (3.1%)	<0.001
Stroke	104 (5.2%)	81 (10.1%)	126 (10.4%)	0.850
Structural Heart disease				
HF	400 (19.9%)	130 (16.3%)	270 (22.3%)	0.001
Prior AMI	158 (7.8%)	85 (10.6%)	73 (6%)	<0.001
AVD	157 (7.8%)	56 (7.0%)	101 (8.3%)	0.277
MVD	221 (11%)	68 (8.5%)	153 (12.6%)	0.004
DCM	71 (3.5%)	49 (6.1%)	22 (1.8%)	<0.001
ICM	184 (9.1%)	89 (11.1%)	95 (7.8%)	0.012
HCM	95 (4.7%)	40 (5%)	55 (4.5%)	0.630
LVEF	49.43 ± 13.43	46.0 ± 14.7 (*n* = 97)	52.4 ± 11.4 (*n* = 111)	0.001
Prior AF known				
AF (all types)	876 (43.52%)	316 (39.5%)	560 (46.2%)	0.003
Paroxysmal	216 (10.7%)	78 (9.8%)	138 (14.4%)	0.341
Persistent	123 (6.1%)	48 (6,2%)	75 (6%)	0.478
Permanent	204 (10.1%)	73 (9.1%)	131 (10.8%)	0.344
CHA_2_DS_2_-VASc score				
Average		2.7 ± 1.6	4.2 ± 1.4	<0.001
HASBLED Scale				
Average		2.5 ± 1.14 (*n* = 533)	2.6 ± 1.01 (*n* = 332)	0.578

Abbreviations: AF = atrial fibrillation; AMI = acute myocardial infarction; AVD = aortic valve disease; CKD = chronic kidney disease; COPD = chronic obstructive pulmonary disease; DCM = dilated cardiomyopathy; HCM = hypertrophic cardiomyopathy; HF = heart failure; HTN = arterial hypertension; ICM = ischemic cardiomyopathy; LVEF = left ventricular ejection fraction; MVD = mitral valve disease; OSA = obstructive sleep apnea; PAD = peripheral artery disease; T2DM = type 2 diabetes mellitus.

**Table 2:** Clinical presentation at emergency room admission by sex group.

	Male (*n* = 800)	Female (*n* = 1213)	*p*-Value
Asymptomatic	72 (9%)	63 (5.3%)	0.007
Chest pain	162 (20.3%)	281 (23.2%)	0.122
Palpitations	202 (25.3%)	406 (33.5%)	<0.001
HF	209 (26.1%)	401 (33.1%)	0.001
APE	25 (3.1%)	61 (5%)	0.039
Dizziness	131 (16.4%)	165 (13.6%)	0.086
Asthenia	47 (5.9%)	94 (7.7%)	0.107
Syncope	39 (4.9%)	52 (4.3%)	0.534
Hemodynamic instability	11 (1.4%)	31 (2.6%)	0.070
HR at admission	117.69 ± 34.665	122.41 ± 31.333	0.002

Abbreviations: APE = acute pulmonary edema; HF = heart failure; HR = heart rate.

**Table 3:** Cox multivariate analyses of risk factors for HF-related hospitalizations.

	B	SE	Wald	*p*-Value	HR	95.0% CI for Exp(B)	
						Lower	Upper
Gender (Female)	0.297	0.118	6.287	0.012	1.346	1.067	1.697
Age	0.017	0.006	9.272	0.002	1.017	1.006	1.028
CKD	−0.310	0.151	4.222	0.040	0.733	0.546	0.986
AMI	0.394	0.161	5.959	0.015	1.483	1.081	2.035
Previous HF	0.561	0.118	22.460	0.000	1.753	1.390	2.211
DCM	0.563	0.209	7.260	0.007	1.756	1.166	2.645
HCM	0.474	0.188	6.343	0.012	1.607	1.111	2.324
CHA2DS2-VASc	0.094	0.043	4.729	0.030	1.099	1.009	1.196
Mitral regurgitation	0.509	0.130	15.250	0.000	1.664	1.289	2.148

Abbreviations: AMI = acute myocardial infarction; CKD = chronic kidney disease; CI = confidence interval; DCM = dilated cardiomyopathy; HCM = hypertrophic cardiomyopathy; HF = heart failure; HR = hazard ratio; SE = standard error; T2DM = type 2 diabetes mellitus.

**Table 4:** Multivariate analyses of age, sex, and comorbidities as risk factors for all-cause mortality.

						95.0% CI for Exp(B)	
	β	SE	Wald	*p*-Value	HR	Lower	Upper
Age (>75) Gender	1.545	0.100	239.132	<0.001	4.689	3.855	5.703
	−0.112	0.078	2.032	0.154	0.894	0.767	1.043
HTN	−0.121	0.091	1.779	0.182	0.886	0.741	1.059
T2DM	0.201	0.073	7.503	0.006	1.223	1.059	1.412
CKD	0.406	0.094	18.453	<0.001	1.501	1.247	1.806
COPD	0.528	0.108	23.973	<0.001	1.695	1.372	2.093
PAD	0.018	0.146	0.015	0.904	1.018	0.764	1.356
AMI	0.405	0.120	11.493	0.001	1.500	1.186	1.895
Stroke	0.438	0.099	19.478	<0.001	1.550	1.276	1.882
HF	0.394	0.083	22.626	<0.001	1.483	1.261	1.744
DCM	0.383	0.165	5.378	0.020	1.467	1.061	2.028

Abbreviations: AMI = acute myocardial infarction; CKD = chronic kidney disease; CI = confidence interval; COPD = chronic obstructive pulmonary disease; DCM = dilated cardiomyopathy; HF = heart failure; HR = hazard ratio; HTN = arterial hypertension; PAD = peripheral artery disease; SE = standard error; T2DM = type 2 diabetes mellitus.

## Data Availability

The datasets used during this study are available from the corresponding authors upon reasonable request.

## Supplementary Materials

The following supporting information can be downloaded at: https://www.mdpi.com/article/10.3390/jcdd10100434/s1, Table S1: Antiarrhythmic and diuretic treatment at admission in the emergency department according to gender; Table S2: Antiarrhythmic and diuretics treatment at discharge from the emergency department according to gender.
