# Supplementary material for: Gender-Based Clinical, Therapeutic Strategies and Prognosis Differences in Atrial Fibrillation
Source: J Cardiovasc Dev Dis. 2023 Oct 18;10(10):434. doi: 10.3390/jcdd10100434 (PMC10607185; doi:10.3390/jcdd10100434)
Supplement: Supplementary file 1 [file jcdd-10-00434-s001.zip › jcdd-2587285-supplementary.pdf]

SUPPLEMENTARY DATA

Table S1. Antiarrhythmic and diuretic treatment at admission in the emergency department according to gender.

	Total (N=2013)	Males (N=800)	Females (N=1213)	p-value
**Amiodarone**	154 (7.7%)	66 (8.3%)	88 (7.3%)	0.411
**Flecainide**	91(4.5%)	24 (3.0%)	67 (5.5%)	0.008
**ß-blockers**	608 (30.2%)	219 (27.4%)	389 (32.1%)	0.025
**Digoxin**	142 (7.0%)	54 (6.8%)	98 (8.1%)	0.269
**Diuretics**	875 (43.5%)	280 (35.0%)	595 (49%)	0.05*

Table S2. Antiarrhythmic and diuretics treatment at discharge from the emergency department according to gender.

	Total (N=2013)	Males (N=800)	Females (N=1213)	p-value
**Amiodarone**	317 (15.7%)	136 (17%)	181 (14.9%)	0.210
**Flecainide**	78 (3.9%)	33 (4.1%)	45 (3.7%)	0.637
**ß-blockers**	859 (42.7%)	322 (40.2%)	537 (44.3%)	0.074
**Digoxin**	227 (11.3%)	69 (8.6%)	158 (13%)	0.002*
**Diuretics**	604 (30%)	206 (25.7%)	398 (32.8%)	0.001*
